# Expression Analysis of Selected microRNAs in Diabetes Mellitus Visceral Fat Tissues

**DOI:** 10.1155/jobe/3230015

**Published:** 2026-04-10

**Authors:** Rabeah Al-Temaimi, Rasheed Ahmad, Kusum Kapila, Fahd Al-Mulla

**Affiliations:** ^1^ Department of Pathology, College of Medicine, Kuwait University, P. O. Box 24923 Jabriya, Safat, 13110, Kuwait, kuniv.edu; ^2^ Department of Immunology & Microbiology, Dasman Diabetes Institute, P.O. Box 1180 Dasman, Sharq, 15462, Kuwait, dasmaninstitute.org; ^3^ Department of Translational Research, Dasman Diabetes Institute, P.O. Box 1180 Dasman, Sharq, 15462, Kuwait, dasmaninstitute.org

**Keywords:** adipocyte, microRNA, RKIP, Type 2 diabetes, visceral fat

## Abstract

**Purpose:**

MicroRNA (miRNA) profiling of visceral adipose tissue in Type‐2 diabetes mellitus (T2DM) remains limited. We compared the expression of obesity‐associated miRNAs in visceral fat from individuals with T2DM versus metabolically healthy obesity (MHO) and examined Raf kinase inhibitory protein (RKIP) as a candidate miR‐543 target.

**Methods:**

Visceral fat biopsies were obtained from adults with T2DM (*n* = 8) and MHO (*n* = 11). Thirteen miRNAs previously linked to obesity were quantified, and RKIP expression was evaluated at the mRNA level and by immunohistochemistry, including phosphorylated RKIP (pRKIP).

**Results:**

Of the 13 miRNAs analyzed, miR‐23a‐3p and miR‐543 were upregulated in T2DM (*p* = 0.032 and *p* = 0.009, respectively), whereas miR‐320a‐3p was downregulated (*p* = 0.009). RKIP mRNA levels did not differ between groups; however, in MHO adipocytes, RKIP mRNA correlated positively with miR‐543 expression (*r* = 0.655, *p* = 0.034). Total RKIP protein was comparable between groups, while pRKIP was significantly higher in T2DM adipocytes (*p* = 0.012).

**Conclusions:**

Posttranscriptional regulation in visceral adipocytes differs between T2DM and MHO. Increased miR‐543 and elevated pRKIP in T2DM suggest a rapid shift in regulatory signaling consistent with enhanced lipolysis and a proinflammatory milieu. Further studies are warranted to delineate the pRKIP‐associated pathways in adipose tissue remodeling in T2DM.

## 1. Introduction

Type‐2​ diabetes mellitus (T2DM) is a chronic, complex metabolic disorder characterized by insulin resistance and consequent hyperglycemia. One of the principal risk factors for T2DM is obesity. Being obese or overweight is associated with excess fat accumulation that predisposes to insulin resistance. Adipose tissue or fat elicits a low‐grade inflammatory immune response as it accumulates, as demonstrated in animal models and human samples [1, 2]. The mechanism by which adipose tissue’s low‐grade inflammation in obese individuals causes insulin resistance is unclear since fat tissue inflammation is a normal physiological process that mediates fat tissue remodeling in response to fluctuating energy expenditure [3]. Paradoxically, blocking inflammation in mouse fat tissue triggered insulin resistance [4]. Fat resection or removal alone does not improve insulin sensitivity [5], but bariatric surgery that influences eating habits and reduces weight gradually has successfully reversed T2DM [6]. Moreover, adipose tissue−derived exosomes and fat‐associated macrophage exosomes from obese animal models have been shown to induce insulin resistance [7]. Exosome cargo includes various macromolecules, including microRNAs (miRNAs), that have the potential to regulate gene expression in the exosome‐receiving cells. miRNAs are short, single‐stranded, noncoding ribonucleic acid molecules that regulate gene expression in the cytoplasm. Since their discovery, miRNAs have been explored in various tissues and diseases, including T2DM and its comorbidities [8–10]. Many circulating and exosome miRNAs have been reported to be altered in T2DM with potential applications as diagnostic biomarkers for prediabetes [11], predictive biomarkers for T2DM complications [12, 13], and markers for response to T2DM treatments [14]. However, few studies have investigated miRNAs that regulate inflammation within diabetic visceral fat cells due primarily to the invasive nature of tissue procurement [15, 16]. Compared to subcutaneous fat, visceral fat tissues are more metabolically active, and the chronic low‐grade inflammation preluding insulin resistance in T2DM is thought to arise in visceral fat [17, 18]. To add to existing evidence, we selected 13 miRNAs that have been found altered in inflammatory conditions, including obesity and T2DM, and assessed their expression in diabetic obese and metabolically healthy obesity (MHO) visceral fat biopsies. We chose MHO visceral fat biopsies instead of lean to control for white adipocyte−associated miRNAs that are expressed primarily to maintain white adipocytes. The miRNAs were chosen based on previously reported functions in regulating inflammation and altered adipose tissue metabolism. In addition, we selected the most statistically significant miRNA altered in diabetic visceral fat and analyzed its reported target in fat biopsies from diabetic and nondiabetic obese or MHO individuals.

## 2. Methods

### 2.1. RNA Extraction From Formalin‐Fixed Paraffin‐Embedded (FFPE) Fat Biopsies

This study included 11 MHO individuals’ visceral fat biopsies and 8 individuals with diabetes visceral fat biopsies. Fat biopsy collection for this study was approved by the Dasman Diabetes Institute’s Ethical Review Committee, which follows the Declaration of Helsinki: ethical principles for medical research involving human subjects (RA 174). The original participant recruitment included a random approach for consent from individuals with obesity with or without T2DM undergoing sleeve gastrectomy. Inclusion criteria for this study were as follows: individuals with obesity (BMI > 30 kg/m^2^) undergoing sleeve gastrectomy, with or without a diagnosis of diabetes mellitus, and being of age > 18 years. Exclusion criteria included individuals suffering from other serious diseases such as cardiovascular, renal, liver, hematologic, or lung disease and individuals with malignancy or immune disorders. In total, 19 participants fulfilled this study’s criteria. Written informed consent was collected from all participants prior to sample collection and preservation as FFPE tissue blocks. Visceral fat issues were collected intraoperatively from the omental visceral fat depot through the standard laparoscopic access used for the bariatric procedure. FFPE fat biopsy tissue blocks were sectioned into seven 10 μm sections each. Sections were collected in a microcentrifuge tube and deparaffinized in 320 μL of xylene. The suspension was vortexed for 10 s and spun down briefly to pellet. The pellet was incubated in xylene at 56°C for 3 min and cooled at room temperature. Qiagen’s miRNeasy FFPE kit (Qiagen, MD, USA) was used to extract miRNA. In brief, 240 μL of PKD buffer was added and mixed by vortexing for 15 s. This was followed by centrifugation at 10,000 rpm for 1 min at room temperature. Ten microliters of proteinase K were added and mixed gently by pipetting. The lysate was incubated at 56°C for 15 min, followed by an incubation at 80°C for 15 min. All incubations had interrupted mixing by vortexing every 5 min. The lower clear phase was transferred to a fresh 2 mL microcentrifuge tube and chilled on ice for 3 min. The samples were centrifuged at 13,500 rpm for 15 min, and the supernatant was transferred without disturbing the debris pellet to a new microcentrifuge tube. DNase booster buffer was added in a volume equal to a 10th of the supernatant volume, followed by 10 μL of DNase I solution. The sample was mixed by gentle inversions and briefly spun down to collect. The reaction was incubated at room temperature for 15 min. Five hundred microliters of RBC buffer were added, followed by adding 1750 μL of absolute ethanol and mixed by pipetting. The mixture was loaded in kit‐supplied spin columns and centrifuged at 11,000 rpm for 15 s. Columns were washed with 500 μL RPE buffer twice with centrifugation at 11,000 rpm for 15 s and 2 min, respectively. The columns were dried by spinning open‐capped at maximum speed for 5 min. Bound miRNA was eluted into a labeled microcentrifuge tube with 25 μL of elution buffer and centrifugation at full speed for 1 min. Total RNA extracts were assessed for quantity and quality using a NanoDrop spectrophotometer. Due to the limited sample and the degraded nature of FFPE RNA, we utilized the A_260_/A_280_ ratio to determine RNA extract quality rather than RNA quality number (RIN) since mature short miRNAs are more stable than long ribosomal RNA species used to calculate the RIN.

### 2.2. Selection of Candidate miRNAs

A literature search was conducted in December 2022 using PubMed (https://pubmed.ncbi.nlm.nih.gov) with the following keywords “visceral fat miRNA” and “diabetes visceral fat miRNA” and “insulin resistance miRNA.” Resultant articles were scrutinized to select the miRNAs that fit our inclusion criteria. The inclusion criteria were miRNAs expressed in adipocytes not in circulation, miRNAs preferably expressed in visceral fat of obese individuals, and miRNAs with validated functional evidence in inflammation or metabolism, and priority was given to miRNAs altered in obese versus insulin‐resistant obese or diabetic obese adipocytes. A technical inclusion condition was the assay availability at selected vendors to not cause assay bias when using custom or alternate vendor assays that may have different performance efficiencies. Exclusion criteria included miRNAs expressed in circulation, miRNAs differentially expressed between lean and obese adipocytes, miRNAs investigated in subcutaneous fat tissues, miRNAs of altered expression in obese fat tissues that do not have an experimentally validated functional evidence, and miRNAs resultant from pooled microarray data without single miRNA assay confirmation. A total of 21 miRNAs were selected, of which 13 were prioritized as they had replicated reported functional evidence. Table 1 lists the 13 miRNAs investigated in this study.

**TABLE 1:** MiRNAs were selected for investigation based on their reported function or association with inflammation and altered metabolism.

MicroRNA	Altered expression	Reference
miR‐23a‐3p	Downregulated in obese and diabetic adipose tissues	[19]
miR‐34a‐3p	Upregulation promotes inflammation in adipose tissues	[20]
miR‐146a‐5p	Upregulation suppressed inflammation in adipose tissues	[21]
miR‐150–5p	Upregulation modulates the inflammatory response in adipose tissues	[22]
miR‐155–5p	Proinflammatory in adipose tissues	[23]
miR‐155–3p	Potential role in immune modulation	[24]
miR‐196a‐3p	Regulates fat distribution	[25]
miR‐221–3p	Differentially expressed in obesity and diabetes adipocytes	[26]
miR‐223–3p	Potential marker for adipose tissue dysfunctional metabolism	[27]
miR‐320a‐3p	Potential function in glucose and lipid metabolism	[28]
miR‐342–3p	Upregulated in obesity and potential role in appetite regulation	[29]
miR‐484	Potential role in insulin resistance	[30]
miR‐543	Potential role in insulin resistance	[31]

### 2.3. miRNA Expression Assays

For the detection and quantification of mature miRNAs in the samples, TaqMan Advanced miRNA cDNA Synthesis Kit (ThermoFisher Scientific, MA, USA) was used according to the manufacturer′s protocols. In brief, the kit requires the addition of a 3′ poly‐A tail to short miRNAs, followed by adapter ligation at the 5′ end of miRNAs, and finally, using a primer pair specific for poly‐A and adapter sequences to convert captured miRNAs into cDNA (reverse transcription). A final minimal cDNA amplification step was followed to ensure uniform amplification of all miRNAs to detectable levels. Next, real‐time PCR was performed using TaqMan Fast Advanced Master Mix (ThermoFisher Scientific, MA, USA) and a 1:10 dilution of the cDNA template and TaqMan’s human advanced miRNA assay (miR‐23a‐3p assay ID: 478,532_mir, miR‐34a‐3p assay ID: 478,047_mir, miR‐146a‐5p assay ID: 478,399_mir, miR‐150–5p assay ID: 477,918_mir, miR‐155–5p assay ID: 477,927_mir, miR‐155–3p assay ID: 477,926_mir, miR‐196a‐3p assay ID: 478,745_mir, miR‐221–3p assay ID: 477,981_mir, miR‐223–3p assay ID: 477,983_mir, miR‐320a‐3p assay ID: 478,594_mir, miR‐342–3p assay ID: 478,043_mir, miR‐484 assay ID: 478,308_mir, and miR‐543 assay ID: 478,155_mir). The real‐time PCR protocol was conducted according to the manufacturer’s instructions. miR‐191–5p (assay ID: 477,952_mir) was used as an endogenous control since it is consistently expressed in adipose tissues to maintain white adipocyte phenotype [32, 33]. In brief, each reaction contained 5 μL of TaqMan Fast Advanced Master Mix, 0.5 μL of TaqMan advanced miRNA assay, 2 μL of RNase‐free water, and 2.5 μL of 1:10 diluted cDNA template. All reactions were run in triplicate for MHO and diabetic visceral fat cDNA samples for 40 cycles, except for miR‐196a‐3p, which was run for 42 cycles to capture two late amplification cycles. Reactions were run on QuantStudio 3 Real‐Time PCR System (Applied Biosystems, CA, USA), and the delta‐delta Ct method (2–ΔΔCt) was used to determine the relative fold expression of selected miRNA. miRNAs’ cycle threshold (Ct) ranges can be viewed in Supporting Table 1.

### 2.4. Raf Kinase Inhibitory Protein (RKIP) mRNA Expression

Real‐time PCR was performed on RNA extracted using the Qiagen RNeasy FFPE kit (Qiagen, MD, USA) according to the manufacturer′s protocol without modification. To determine the mRNA expression of the RKIP in fat biopsies, TaqMan Fast Virus 1‐Step Master Mix (ThermoFisher, MA, USA) was used to convert mRNA into cDNA and assess expression in a single real‐time PCR run according to the manufacturer’s protocol. TaqMan RKIP (Hs00831506_g1) and eukaryotic 18S rRNA (X03,205.1) expression assays (ThermoFisher Scientific, MA, USA) were used. Reaction plates were run on QuantStudio 3 Real‐Time PCR System (Applied Biosystems, CA, USA). The fold expression of RKIP was analyzed using the comparative Ct (2^–ΔΔCt^) method.

### 2.5. Immunohistochemistry

Human PEBP1 polyclonal antibody (ID: PA5‐145417, Invitrogen, MA, USA) and human phospho‐RKIP (Ser153) polyclonal antibody (ID: BS‐7075R, BIOSS, Inc., MA, USA), both raised in rabbit, were used to determine the protein expression of RKIP in fat biopsies and pancreatic adenocarcinoma fine needle aspirates (FNAs) cell block tissues as reference for disrupted RKIP expression. In brief, tissue sections were mounted on slides and deparaffinized in xylene for 5 min three times at room temperature. Rehydration was performed in a descending ethanol concentration series for 3 min in each concentration. Slides were washed with deionized water and quenched in 3% hydrogen peroxide for 10 min. Slides were rinsed and heated to induce epitope retrieval by submerging in 0.1 M citrate buffer (pH 6.0) at 100°C for 20 min in a microwave oven. After cooling, slides were kept in Tris‐buffer saline (TBS) (pH 7.6) for 5 min, followed by incubation in 10% goat serum for 30 min to block any reactive antigens in tissues. This was followed by incubation with primary antibodies diluted to their recommended dilutions in 1% goat serum TBS at 4°C overnight. The next day, slides were washed in TBS for 3 min three times and incubated for 30 min with EnVision + peroxidase‐conjugated anti‐Rabbit IgG secondary antibodies (Agilent, Santa Clara, CA, USA). Following incubation, the slides were washed with three changes of TBS, and DAB substrate was added and incubated for 2 min at room temperature to allow color formation. Slides were washed under tap water for 5 min, followed by counterstaining with hematoxylin for 30 s and rehydration in an ascending ethanol series. Slides were cleared of residual ethanol and water by washing three times in xylene for 3 min each, and coverslips were mounted using DPX mounting medium and left to dry for 15 min at room temperature.

### 2.6. Data Analysis

MiRNA and RKIP expression analysis was performed using the relative gene expression or comparative Ct method. Given the small sample size, only nonparametric statistical analyses were performed. Immunohistochemically stained sections were scored for abundance and intensity according to the following: negative (0: no staining), weakly positive (1+: pale orange granular), moderately positive (2+: orange), and strongly positive (3+: dark brownish orange). Spearman correlation was used to identify the relationship between miR‐543 and RKIP mRNA expressions. All analyses used GraphPad Prism (GraphPad Software, MA, USA). A *p* value < 0.05 was considered significant, and no adjustment was performed to the significance level due to the small sample size and the limited number of statistical analyses performed. However, it should be noted that this may increase the risk of Type‐I error.

## 3. Results

### 3.1. MiRNA Expression in Fat Biopsies

Our study consisted of 19 visceral fat biopsies, of which 8 were from individuals with diabetes. The two groups’ demographics and clinical characteristics are shown in Table 2. The expression of 13 miRNAs was assessed in visceral fat biopsies’ total RNA extracts. Among the 13 miRNAs assessed, miR‐155–3p was undetectable in most samples except for 1 diabetic fat biopsy. The remaining miRNAs were detectable in all samples, with few exceptions (Table 3).

**TABLE 2:** Individuals with diabetes and metabolically healthy obesity participants’ demographics and clinical characteristics.

Characteristic/variable	Individuals with metabolically healthy obesity (*n* = 11)	Individuals with diabetes (*n* = 8)	*p* value
Sex assigned at birth			
Male	3 (27.3)	2 (25.0)	0.07
Female	8 (72.7)	6 (75.0)	
Age in years	37 (21–46)	44 (31–52)	0.23
Body mass index	43.25 (39.8–45.3)	40.1 (32.2–44.8)	0.31
Glucose (mmol/L)	5.1 (4.8–6.7)	6.4 (5.6–9.1)	0.033
Urea (mmol/L)	3.3 (1.85–4.65)	3.2 (2.3–8.5)	0.75
Creatinine (umol/L)	61.5 (54.5–72.5)	64 (49–153)	0.98
Phosphate (IU/L)	1.15 (1.1–4.65)	1.18 (0.97–1.26)	> 0.99
Total protein (g/L)	65 (59–69)	75 (64–77)	0.25
Albumin (g/L)	33 (29–39)	38 (34.5–39)	0.55
Urate (umol/L)	324 (271–362.3)	275 (243.5–367)	0.69
Total bilirubin (umol/L)	11 (10–21)	10 (6.75–22.2)	0.61

*Note:* All quantitative variables are presented as median and interquartile range (IQR).

**TABLE 3:** Median raw miRNA fold expression change median values and interquartile range (IQR) of metabolically healthy obese fat (MHOF) tissues compared to diabetic fat (DF) tissues.

miRNA	Number expressed		Median fold expression (IQR)		*p* value
	MHOF	DF	MHOF	DF	
miR‐23a‐3p	11	8	1.1 (0.8–2.2)	2.2 (1.6–3.0)	**0.020**
miR‐34a‐3p	11	8	1.1 (0.6–2.0)	5.8 (0.7–8.6)	0.064
miR‐146a‐5p	10	8	0.8 (0.4–1.7)	1.0 (0.7–1.3)	> 0.999
miR‐150–5p	11	8	0.5 (0.3–1.5)	0.7 (0.5–1.0)	0.967
miR‐155–5p	4	8	0.4 (0.2–1.0)	1.3 (0.2–3.0)	0.537
miR‐196a‐3p	4	7	1.0 (0.5–2.6)	0.4 (0.2–1.2)	0.527
miR‐221–3p	11	8	1.0 (0.4–3.5)	1.4 (0.7–1.8)	0.599
miR‐223–3p	11	8	0.8 (0.6–1.2)	2.1 (0.7–3.5)	0.227
miR‐320a‐3p	11	8	0.9 (0.8–1.5)	0.2 (0.3–0.6)	**0.009**
miR‐342–3p	11	8	0.8 (0.5–1.5)	1.1 (0.7–1.6)	0.544
miR‐484	11	8	1.0 (0.5–2.9)	2.8 (1.3–3.4)	0.151
miR‐543	11	8	0.7 (0.4–1.2)	3.6 (2.7–7.2)	**0.009**

*Note:* *p* values are from the Mann—Whitney U test statistical analysis. Statistically significant p values are highlighted in bold.

**FIGURE 1:**
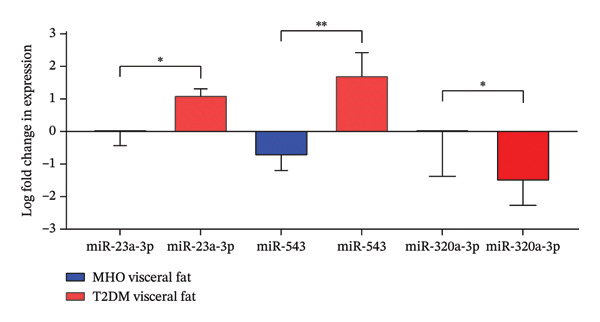
Log‐transformed fold change in expression of miRNAs that show significant differential expression in metabolically healthy obese (MHO) compared to diabetic visceral fat tissues. ^∗^Degree of statistical significance.

Log‐transformed fold expression showed significant associations in agreement with raw miRNA fold expression association results (Figure 1). miR‐23a‐3p and miR‐543 log‐transformed fold expression were significantly overexpressed in diabetic adipocytes than in MHO (*p* = 0.02 and 0.0054, respectively). Meanwhile, miR‐320a‐3p was significantly downregulated in diabetic adipocytes compared to MHO adipocytes (*p* = 0.011). The miRNA with the most significantly different expression between MHO and diabetic visceral fat samples was miR‐543.

### 3.2. RKIP Expression in Adipocytes

A prominent target of miR‐543 is the RKIP, which plays a pivotal role in inflammatory pathways. We assessed the mRNA levels of *RKIP* in the visceral fat groups and found its expression was similar in the two groups (*p* > 0.05). However, RKIP expression correlated with miR‐543 in the MHO visceral fat tissues (*r* = 0.655, *p* = 0.034). In contrast, in diabetic visceral adipocytes, no correlation was found. RKIP is present in cells in native and phosphorylated forms, each activating specific pathways; therefore, we investigated the protein expression of RKIP and phosho‐RKIP (pRKIP) in MHO and diabetic visceral adipocytes. We found RKIP expressed at comparable levels in the plasma membrane and cytoplasm in MHO and diabetic adipocytes (Figure 2(a)). In contrast, pRKIP expression was positive in most specimens except for one MHO fat biopsy. pRKIP expression was expressed in the cytoplasm at varying intensities, predominantly mild to sparse (+1, 63.6%) in MHO visceral fat specimens. In diabetic visceral fat specimens, pRKIP maintained its cytoplasmic expression; however, 25% of diabetic fat specimens showed concurrent membranous and cytoplasmic pRKIP expression. Moreover, pRKIP intensity was higher in diabetic fat specimens than MHO fat specimens (*p* = 0.012, Figure 2(b)). pRKIP was moderate to high in 75% of diabetic visceral fat specimens. In subcutaneous fat, RKIP and pRKIP expression did not differ between the two groups.

FIGURE 2Visceral fat biopsy tissues RKIP and pRKIP immunohistochemistry. (a) The upper panel shows RKIP staining in healthy, obese visceral fat tissues at different magnifications. The lower panel shows RKIP staining in diabetic visceral fat tissue. (b) The upper panel shows pRKIP staining in metabolically healthy obese visceral fat tissue, while the lower panel shows pRKIP staining in diabetic visceral fat tissue. Images were captured at ascending magnifications of 200X, 400X, and 600X magnification, and scale bars denote 100, 20, and 10 μm, respectively.

**Figure:**
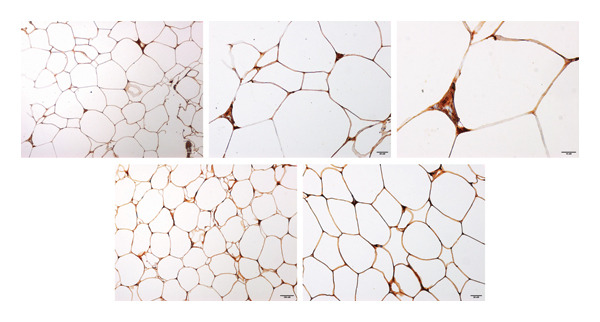
(a)

**Figure:**
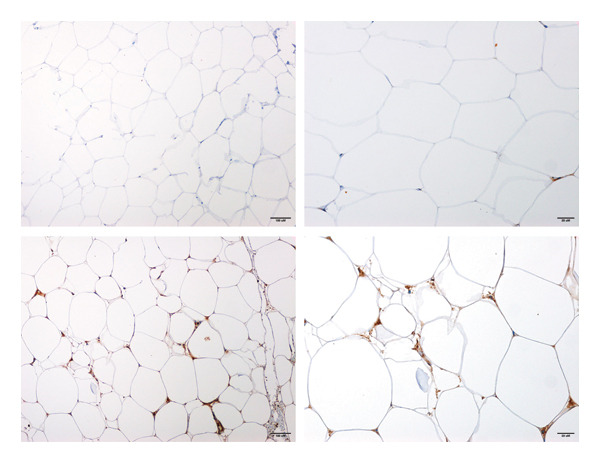
(b)

## 4. Discussion

When visceral fat increases beyond 10% of total body fat, it predisposes to metabolic abnormalities and persistent low‐grade inflammation, which in turn results in insulin resistance and T2DM [34]. Here, we collected visceral fat biopsies from MHO (nondiabetic) individuals and obese individuals with diabetes to explore multiple miRNAs that may play a role in the pathogenic shift from MHO to diabetic obese. Several of the miRNAs we explored have already been analyzed in different diabetic individuals’ samples and obese individuals, as listed in Table 1. However, our aim was to delineate the critical miRNAs that are associated with the mechanistic metabolic and inflammatory alterations mediating T2DM pathogenesis. Three miRNAs were significantly differentially expressed between obese T2DM and MHO individuals’ visceral fat, namely, miR‐23a‐3p, −320a‐3p, and −543. In the case of miR‐23a‐3p, our result is contrary to reports of its decrease in T2DM [19, 35]. In those reports, serum samples from individuals with T2DM were used, whereas we investigated visceral fat. Conversely, in gestational diabetes, miR‐23a‐3p was found increased in plasma samples [36]. The proposed role of miR‐23a‐3p has been investigated in several disease models, including cancer [37], autoimmune diseases [38], and obesity [19]. A prominent role that may explain its increase in diabetic visceral fat is its involvement in inflammation and immune regulation [39, 40]. Mir‐320a‐3p, on the other hand, was reduced in diabetic visceral fat biopsies in our cohort, but previous reports have shown it to be increased in diabetic sera as well as in other inflammatory conditions [28]. Aside from tissue type differences, most of those reports fail to define which isotype (a, b, c, d, or e) or mature miRNA strand (5p or 3p) was being assessed. Assessing the unprocessed miR‐320 may likely result in an inaccurate designation of functional mature copies as biomarkers for specific diseases studied. Lastly, we found that miR‐543 was high in diabetic visceral fat biopsies and had the most significant association with T2DM. While many studies investigated miR‐543 in different cancers [41], only a few investigated its role in T2DM and its comorbidities [42, 43]. Of note is a study that investigated public T2DM miRNA datasets and found miR‐543 to be the most significantly elevated miRNA [44]. It also suggested its potential as a novel biomarker for T2DM, possibly paving the way for innovative treatments. Among the 13 miRNAs we investigated, 10 previously reported to associate with T2DM or altered metabolism failed to show statistical associations. However, miR‐155–5p was only detectable in 4 MHO individuals, of which 3 showed a reduction in its level. The MHO individual who showed an increase had the highest BMI in the total MHO cohort (49.45), suggesting that he may be in the prediabetes stage. Therefore, for miR‐155–5p, we cannot refute recent evidence of its potential as a diagnostic biomarker for T2DM risk in obese individuals [45, 46].

An evidence‐backed target of miR‐543 is RKIP, which plays a role in multiple pathways, most prominently inflammation [47]. RKIP’s role as a tumor suppressor gene is well established, and its involvement in the negative regulation of NLRP1, NLRP3, and NLRC4 inflammasomes has been shown [48]. RKIP expression has never been investigated in T2DM adipose tissues. We found RKIP mRNA expression to be comparable in both MHO and obese diabetic visceral fat biopsies. However, its increased expression directly correlated with miR‐543 expression in the MHO group but not the diabetic obese visceral fat. This suggests that the regulatory mechanism of RKIP expression is altered in diabetic visceral fat, whereas in MHO visceral fat, RKIP expression epigenetic regulation is maintained. Moreover, RKIP protein expressions were comparable in the two groups. However, the expression of pRKIP was significantly higher in diabetic visceral fat than in MHO visceral fat. Phosphorylation of RKIP at Serine residue 153 allows it to bind G‐protein coupled receptor (GPCR) kinase 2 (GRK2) to activate several pathways, including the stimulation of ERK1 activity to promote adipogenesis, and the promotion and activation of inflammatory signaling pathways, including the NF‐κB pathway, the STAT‐3 pathway, as well as the regulation of the GSK3 beta [47]. These proinflammatory pathways have been shown to be activated in T2DM, and RKIP’s involvement has been proposed [49–51]. However, no studies have been conducted to determine RKIP or pRKIP levels in diabetic adipose tissues to confirm or refute such associations. Here, we show that not only RKIP epigenetic regulation is disrupted in diabetes but also pRKIP is predominantly activated in diabetes to promote the pivotal pathways of T2DM pathogenesis, namely, the ERK1/2, NF‐κB, and STAT‐3 pathways. pRKIP mediates the activation of these pathways after dissociating from Raf1 and binds to GRK2, releasing GPCRs from it and activating its downstream pathways [52]. GPCRs regulate most metabolic processes in multiple tissues and have been extensively researched for their roles in obesity and T2DM [53, 54]. GPCRs have also been suggested as potential therapeutic targets for T2DM, albeit with limited progress so far [55]. In adipocytes, different GPCRs have been shown to have different biological effects when bound to various ligands [53]. Moreover, pRKIP expression in visceral fat may allow adrenergic GPCRs to induce lipolysis when bound to catecholamines and acetylcholine [56, 57]. In obesity, lipolysis is increased, contributing to the release of excess fatty acids, perturbing insulin action, and promoting inflammation that is suggested to prelude insulin resistance [58]. Therefore, reducing the level of RKIP phosphorylation may offer a potential therapeutic benefit by minimizing the effect of GPCR‐induced lipolysis and the activation of proinflammatory pathways.

A major limitation in our study is the sample size, which is very small and may lessen the statistical power of our analysis, stressing the need for larger cohort studies. However, visceral fat biopsies are difficult to collect due to their invasive nature, and patient consent was rare. While subcutaneous fat might be easier to acquire, we believe visceral fat plays a critical role in T2DM inflammation compared to subcutaneous fat and is more valuable in determining T2DM pathogenesis. Another limitation was the use of FFPE tissue biopsies that may have affected the quality of cellular miRNA pools. However, miRNAs are very stable RNA species due to their small size and have been shown repeatedly to have enhanced stability under various fixation conditions [59, 60]. In addition, we did not correct for multiple testing due to the small sample size and the exploratory nature of our study, and as such, our results may be subject to Type‐I error. Lastly, our chosen miRNA panel may be restricted to previous literature, despite our replicated findings of recent studies and novel findings. Recent studies have highlighted several miRNAs of interest that may be involved in the pathogenesis of T2DM. For example, an RNA sequencing study reported novel differentially expressed miRNAs associated with obese T2DM compared to MHO, namely, miR‐203a‐3p, miR‐216a‐5p, miR‐30c‐2‐3p, miR‐5584–5p, and miR‐184 [61]. In addition, a study on T2DM visceral fat tissues compared to normal glycemic and impaired glycemic visceral fat tissues analyzed a set of 18 miRNAs predicted to regulate major genes involved in oxidative stress, glucose metabolism, and inflammation and found 5 to be altered in T2DM [16]. While it would have been valuable to include these miRNAs along with others in our study, it should be noted that most of the recent reports investigated circulating miRNAs for their T2DM diagnostic potential or their prediction of T2DM comorbidities rather than T2DM pathogenesis in visceral fat tissues [62, 63]. Moreover, recent reports predominantly included normal controls rather than matched MHO, whereas our study focused on miRNAs potentially involved in T2DM pathogenesis in visceral fat biopsies of obese individuals in comparison to MHO individuals. In conclusion, we have shown that miR‐23a‐3p, miR‐320a‐3p, and miR‐543 expressions are altered in diabetic visceral fat compared to MHO visceral fat. However, miR‐543 expression did not show any correlation with its known target RKIP, neither on mRNA nor protein expression levels. Moreover, we found pRKIP to be at least two‐fold increased in diabetic obese visceral fat when compared to healthy obese visceral fat. Future studies will focus on assessing which GPCR is most affected by this increase and whether they offer new insight into T2DM pathogenesis in adipose tissues.

## Author Contributions

Rabeah Al‐Temaimi: conceptualization, methodology, data collection, formal analysis, and manuscript write‐up. Rasheed Ahmad: sample and associated data collection. Kusum Kapila: imaging and biomarker scoring. Fahd Al‐Mulla: facilitating project tasks and progress and reviewing and editing the manuscript.

## Funding

No funding was received for conducting this study.

## Disclosure

All authors have read and approved the final manuscript.

## Ethics Statement

Ethical approval was granted by the Dasman Diabetes Institute’s Ethical Review Committee (RA 174).

## Consent

All participants in this study provided their written informed consent.

## Conflicts of Interest

The authors declare no conflicts of interest.

## Supporting Information

Supporting Table 1: Cycle threshold (Ct) ranges of each miRNA assessed in visceral fat biopsies.

## Supporting information


**Supporting Information** Additional supporting information can be found online in the Supporting Information section.

## Data Availability

The data that support the findings of this study are available from the corresponding author upon reasonable request.
