# Supplementary material for: Expression Analysis of Selected microRNAs in Diabetes Mellitus Visceral Fat Tissues
Source: J Obes. 2026 Apr 10;2026:3230015. doi: 10.1155/jobe/3230015 (PMC13067198; doi:10.1155/jobe/3230015)
Supplement: Supplementary file 1 — Supporting Information Additional supporting information can be found online in the Supporting Information section. [file JOBE-2026-3230015-s001.docx]

**Supplementary table 1.** Cycle threshold (Ct) ranges of each miRNA assessed in visceral fat biopsies sorted according to tested groups; metabolically healthy obesity individuals’ group and individuals with diabetes.

| **miRNA** | **Individuals with metabolically healthy obesity** | **Individuals with diabetes** |
| --- | --- | --- |
| miR-23a-3p | 23.6 – 28.2 | 21.0 – 25.4 |
| miR-34a-3p | 31.8 – 37.2 | 29.8 – 33.5 |
| miR-146a-5p | 24.4 – 31.2 | 23.2 – 27.6 |
| miR-150-5p | 21.7 – 27.0 | 21.1 – 25.0 |
| miR-155-5p | 28.4 – 32.3 | 22.2 – 34.8 |
| miR-196a-3p | 38.9 – 40.6 | 32.1 – 40.5 |
| miR-221-3p | 21.9 – 27.5 | 21.5 – 24.6 |
| miR-223-3p | 23.4 – 27.9 | 20.1 – 26.7 |
| miR-320a-3p | 23.6 – 29.2 | 24.5 – 27.7 |
| miR-342-3p | 28.0 – 32.4 | 26.1 – 31.1 |
| miR-484 | 24.0 – 31.6 | 25.0 – 27.7 |
| miR-543 | 31.2 – 38.9 | 28.7 – 31.6 |
| miR-191-5p | 23.6 – 28.2 | 22.4 – 27.1 |
